# Fatty Acid Ethyl Esters Induce Intestinal Epithelial Barrier Dysfunction via a Reactive Oxygen Species-Dependent Mechanism in a Three-Dimensional Cell Culture Model

**DOI:** 10.1371/journal.pone.0058561

**Published:** 2013-03-19

**Authors:** Elhaseen Elamin, Ad Masclee, Kati Juuti-Uusitalo, Sven van IJzendoorn, Freddy Troost, Harm-Jan Pieters, Jan Dekker, Daisy Jonkers

**Affiliations:** 1 Top Institute Food and Nutrition (TIFN), Wageningen, The Netherlands; 2 Department of Internal Medicine, Maastricht University Medical Center, Maastricht, The Netherlands; 3 Department of Cell Biology, University Medical Center Groningen, Groningen, The Netherlands; 4 Institute of Biomedical Technology, University of Tampere, Tampere, Finland; 5 Department of Animal Sciences, Wageningen University, Wageningen, The Netherlands; Biological Research Centre of the Hungarian Academy of Sciences, Hungary

## Abstract

**Background & Aims:**

Evidence is accumulating that ethanol and its oxidative metabolite, acetaldehyde, can disrupt intestinal epithelial integrity, an important factor contributing to ethanol-induced liver injury. However, ethanol can also be metabolized non-oxidatively generating phosphatidylethanol and fatty acid ethyl esters (FAEEs). This study aims to investigate the effects of FAEEs on barrier function, and to explore the role of oxidative stress as possible mechanism.

**Methods:**

Epithelial permeability was assessed by paracellular flux of fluorescein isothiocyanate-conjugated dextran using live cell imaging. Cell integrity was evaluated by lactate dehydrogenase release. Localization and protein levels of ZO-1 and occludin were analyzed by immunofluorescence and cell-based ELISA, respectively. Intracellular oxidative stress and cellular ATP levels were measured by dichlorofluorescein and luciferase driven bioluminescence, respectively.

**Results:**

In vitro, ethyl oleate and ethyl palmitate dose dependently increased permeability associated with disruption and decreased ZO-1 and occludin protein levels, respectively, and increased intracellular oxidative stress without compromising cell viability. These effects could partially be attenuated by pretreatment with the antioxidant, resveratrol, pointing to the role of oxidative stress in the FAEEs-induced intestinal barrier dysfunction.

**Conclusions:**

These findings show that FAEEs can induce intestinal barrier dysfunction by disrupting the tight junctions, most likely via reactive oxygen species-dependent mechanism.

## Introduction

Ethanol is widely consumed worldwide and associated with the development of alcoholic liver diseases (ALD). Disruption of tight junctions (TJs) between intestinal epithelial cells may lead to an increased intestinal permeability resulting in an enhanced permeation of toxins and pathogens into the circulation with subsequent endotoxaemia [1]. This process is considered to play a key role in the pathogenesis of ALD [2], [3]. There is increasing evidence that ethanol [4], [5], [6] and to a greater extent, its oxidative metabolite, acetaldehyde [7], [8] can disrupt the TJs and increase paracellular permeability in Caco-2 cell monolayers by mechanisms involving e.g. inducible nitric oxide synthase (iNOS)-mediated reactive oxygen species (ROS) generation [5], [6], and protein tyrosine phosphorylation [9]. A part from oxidative metabolism, ethanol can be metabolized non-oxidatively by the enzyme phospholipase D and fatty acid ethyl ester synthases, generating phosphatidylethanol and fatty acid ethyl esters (FAEEs), respectively [10], [11]. FAEEs including ethyl oleate and ethyl palmitate, have been detected in blood at concentrations ranging between 10 µM and 50 µM with half-life of 24 and 44–99 h following moderate and heavy ethanol consumption, respectively, and have been proposed as biomarkers for both recent and long-term ethanol intake [12], [13], [14]. Increased levels of FAEEs have also been found in the liver, pancreas, heart and adipose tissue following death of intoxicated subjects [15]. Since fatty acids and ethanol are absorbed by enterocytes, the intestine is also considered a major site for FAEEs synthesis [16]. Moreover, duodenal mucosa has been found to possess high FAEE synthase activity with subsequent FAEEs production [16]. However, little is known about concentrations present in the intestine after ethanol ingestion.

FAEEs have been shown to accumulate in the hydrophobic membranes resulting in uncoupling oxidative phosphorylation and fragility of the mitochondria and lysosomes, respectively [17], [18]. Moreover, FAEEs can decrease protein synthesis and induce apoptosis [19], [20], [21]. Collectively, these studies strongly suggest that FAEEs may induce cell injury. Although evidence indicates that FAEEs exert cytotoxic activity in pancreatic [18], [22] and liver cells [21], potential effects on intestinal epithelial cells are unknown.

The present study aimed to investigate the effects of FAEEs on intestinal paracellular barrier function by using a three-dimensional (3D) cell culture model of Caco-2 cells [23]. Since metabolic stress can affect the TJs integrity and FAAEs have been demonstrated to inhibit mitochondrial function by uncoupling oxidative phosphorylation [17], the effects of FAEEs on cellular oxidative stress and intracellular ATP levels were also investigated.

## Materials and Methods

### Cell line and culture conditions

Caco-2 cells from the American Type Culture Collection, (ATCC, Rockville, USA) were maintained in Dulbecco's Modified Eagle Medium (DMEM; Lonza Benelux BV, Breda, NL) containing 4.5 g/l glucose and L-glutamine, 10% (v/v) fetal calf serum (Invitrogen, Breda, the Netherlands), 1% (v/v) solution of non-essential amino acids (Invitrogen) and 1% (v/v) solution of antibiotic/antimycotic mixture (10,000 units of penicillin, 10,000 µg of Streptomycin, and 25 µg of Amphotericin B per ml; Invitrogen) in an atmosphere of 5% CO_2_ at 37°C.

### Three dimensional epithelial cell culture

Caco-2 cells were initially grown in growth factor-reduced Matrigel® (8 mg/ml; BD Biosciences, San Jose, California USA) in 10 mm glass bottom culture dishes of 35 mm diameter (MatTek Corporation, Ashland, USA) for barrier function and immunofluorescence analysis, and in 96 well-plates (Corning BV, Amsterdam, the Netherlands) for redox state, cell viability, ELISA cell-based and ATP assays, as described previously[24]. Briefly, The Caco-2 cells (50×10^3^ cells/well; passage 30–38) were resuspended in serum-free medium, mixed with 40% (v/v) Matrigel and plated on the solidified Matrigel. Thereafter, the complete growth medium was added and spheroids were allowed to form at 37°C for 5–7 days. The quality of cultures was checked by counting the number of spheroids and classifying them according to the number of lumens formed. Only cultures containing >70% of spheroids with a single lumen were used for further experiments.

### Exposure to FAEEs and determination of intestinal epithelial barrier function

FAEEs were dissolved in 0.1% dimethyl sulfoxide (DMSO) and spheroids were exposed to either 20 µM or 40 µM of ethyl oleate (EO) or ethyl palmitate (EP) for 24 h [25]. Two mM ethylene glycol tetra acetic acid (EGTA) to induce maximum TJs disruption and growth medium only were used as positive and negative control, respectively. To determine paracellular barrier function, spheroids were incubated with the above indicated concentrations in the presence of 1 mg/ml fluorescein isothothiocyanate-labeled dextran of 4 KDa (FITC-dextran-4; Sigma Chemical Co, Amsterdam, NL) for 24 h. Barrier function was assessed by the flux of FITC-dextran-4 from the basal to the luminal compartment (i.e., L/B ratio) using confocal microscopy. Confocal images were taken with Leica TCS SPE confocal laser scanning microscope (Leica Microsystems GmbH, Mannheim, Germany) and processed using TCS SPE browser and Image J software [26].

### Lactate dehydrogenase assay

Cell plasma membrane integrity was evaluated by measuring lactate dehydrogenase (LDH) release. The assay (CytoTox-ONE™ Homogeneous Membrane Integrity Assay; Promega, the Netherlands) was performed according to the manufacture instructions. Briefly, Caco-2 cells were cultured in 96 well-plates in 3D and incubated with 20 or 40 µM EO or EP. Then, plates were incubated at 37 °C for 24 h and equilibrated at room temperature (RT) for 20 min. Next, 100 µL of the reconstituted substrate mix was added. The plate was incubated at RT, protected from light, for 30 min and thereafter, 20 µL of stop solution was added. Maximum LDH release was induced by using lysis solution. The fluorescence was measured at an excitation and an emission wavelength of 560 nm and 590 nm. The percentage of LDH activity was calculated as percentage of maximum LDH release (i.e. fully lysed cells).

### Assessment of ZO-1 and occludin localization

At the end of FAEEs exposure, Caco-2 spheroids in culture dishes were fixed in 4% (w/v) paraformaldehyde in Hank's Buffered Salt Solution (HBSS; Invitrogen) at 37 °C for 40 min and processed for immunocytochemistry as described previously [24]. Briefly, spheroids were permeabilized with 0.1% (v/v) Triton X-100 in PBS at RT for 40 min and were incubated with a blocking buffer containing 3% (w/v) bovine serum albumin (BSA) in PBS, pH 7.4, at 37°C for 2 h. Spheroids were then incubated overnight with mouse anti-ZO-1 (Zymed Laboratories, San Francisco, USA) and rabbit anti-occludin (Zymed Laboratories) at 1∶100 dilution in 3% (w/v) BSA in PBS, pH 7.4 at 4°C. Next, spheroids were incubated with Alexa-488 conjugated goat anti-mouse (Invitrogen) and or Cy3-conjugated goat anti-rabbit (Jackson Laboratories, Suffolk, UK) secondary antibodies (1∶100 dilution) at 37°C for 1.5 h. After that spheroids were stained for 5 min with diamidino-2-phenylindole (DAPI; 1∶10,000 dilution in PBS; Sigma Chemical Co) and mounted in dishes using VectaShield mounting medium (Vector Laboratories, Burlingame, USA). Confocal images were obtained using a Leica TCS SPE confocal laser scanning microscope. Image J software was used to process and analyze the images [27].

### Assessment of ZO-1 and occludin protein levels

ZO-1 and occludin protein levels were assessed using a cell-based ELISA kits (Ray Biotech, Inc. Norcross, GA, USA) with minor modifications. Briefly, Caco-2 spheroids grown on 96 well-plates (Corning BV, Amsterdam, the Netherlands) were exposed to medium only as control and either 40 µM EO or EP alone for 24 h, or after pretreatment for 1 h with 10 µM of the antioxidant resveratrol (*trans*-3,4′,5-trihydroxy stilbene) [28]. The cultures were washed with PBS and then 100 µl of fixative solution (provided in the kit) was added to each well, and incubated with shaking at RT for 20 min. After another washing, a quenching buffer was added at RT for 20 min, followed by a blocking solution for 1 h at 37°C. After washing thrice, either mouse anti-ZO-1 or rabbit anti-occludin (1∶100 dilution in the blocking solution; Cell Signaling Technology, Inc, MA, USA) was added, and the plate was incubated with shaking at RT for 1 h. Next, 25 µl of HRP-conjugated mouse anti-rabbit IgG (1∶100 dilution in the blocking solution; Dako Netherlands BV, Heverlee, Belgium) was added to each well and incubated at RT for 1 h. Then, the plate was washed three times and 100 µl of 3, 3′, 5, 5′-tetramethylbenzidine (TMB) was added to each well and incubated with shaking in the dark at RT for 30 min. Finally, 25 µl of stop solution was added to each well and the optical density was read at 450 nm with a spectrophotometer.

### Detection of reactive oxygen species (ROS)

The generation of H_2_O_2_ was monitored by using 2′, 7′- dichlorodihydrofluorescein diacetate (DCF-DA, Sigma Chemical Co, Amsterdam, NL), which is non-fluorescent unless oxidized by intracellular reactive oxygen species (ROS). Caco-2 cells were cultured in 96 well plates in 3D and were preloaded with 100 µM DCF-DA for 1 h at 37°C. Dose dependent measurement of the generation of ROS was done by incubating the cells with FAEEs for 24 h. H_2_O_2_ (30 µM) treated spheroids were used as positive control. Spheroids were then washed twice in HBSS buffer and the fluorescence was measured at an excitation and an emission wavelength of 485 nm and 540 nm, respectively.

### Luminescent ATP assay

The number of viable cells in culture was quantified based on the amount of ATP produced by metabolically active cells using luminescent assay kits (CellTiter-Glo® Luminescent Cell Viability Assay, Promega, the Netherlands) according to the manufacture instruction. Briefly, Caco-2 cells were cultured in 96 well-plates in 3D and were incubated with medium only (‘negative’ control), 20 or 40 µM of either EO or EP, and 30 µM H_2_O_2_ (‘positive’ control). Then, plates were incubated at 37 °C for 24 h and equilibrated at RT for 30 min. Thereafter, 100 µl of the reagent assay was added to each well. The contents were mixed on an orbital shaker for 2 min to induce cell lysis and incubated at RT for 10 min to stabilize the luminescent signal. The luminescence was measured using SpectraMax M2 (Molecular Devices, Sunnyvale, CA, USA). ATP level was calculated from the luminescent values and presented as a percentage of the medium only-treated ‘negative’ control.

### Statistical analysis

All experiments were performed in triplicate and results were reported as means±SD of at least 8 spheroids per experiment. A one-way analysis of variance (ANOVA) and Tukey's post hoc test were performed to determine significant differences between experimental conditions. The correlation between intra-cellular ROS and barrier dysfunction was analyzed by using Spearman test. Differences were considered statistically significant when *P*<0.05. All data analyses were conducted with GraphPad Prism software package (GraphPad Software Incorporated, CA, USA).

## Results

### Effects of FAEEs on paracellular permeability

**Figure 1:**
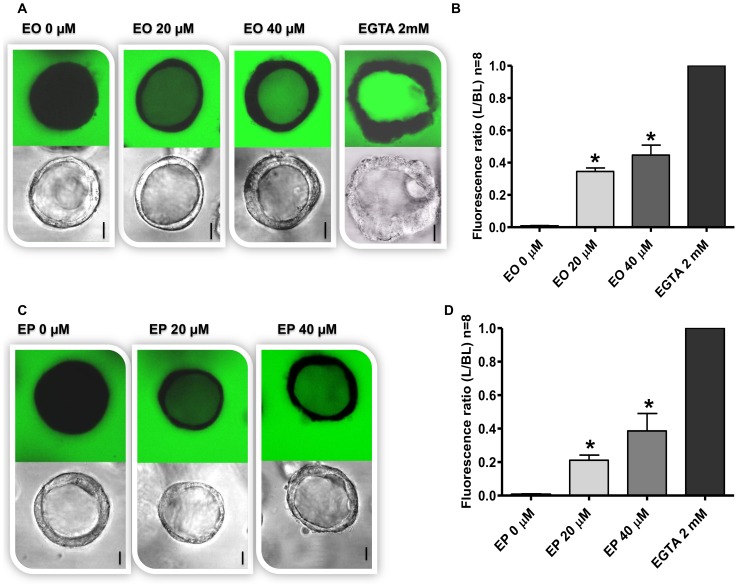
Effect of ethyl oleate (EO) and ethyl palmitate (EP) on paracellular permeability in 3D Caco-2 spheroids. [**A**] Spheroids were treated or not treated with either EO (20, 40 µM), EGTA (positive control) or [**C**] EP (20, 40 µM) in the presence of FITC-dextran-4 at 37 °C for 24 h. Intraluminal accumulation of FITC-dextran-4 (green) was observed using confocal microscopy and representative images captured from the middle of spheroids are shown. The bar indicates 10 µm. The mean fluorescence intensity of FITC-dextran-4 after exposure to [**B**] EO and [**D**] EP was measured and expressed as the ratio of the luminal (L) over the basal (BL) compartment. The L/BL ratio as determined following EGTA exposure was set to 1. All graphs indicate the results of three replicate experiments. Data expressed as means±SD, *p<0.0001, compared to medium only-treated control.

To determine the effect of FAEEs exposure on intestinal epithelium permeability, Caco-2 spheroids were treated with EO and EP for 24 h. In medium-only treated spheroids, FITC-dextran-4 was exclusively observed in the culture medium at the basolateral side of spheroids (Figure 1A) resulting in a very low L/BL fluorescence ratio (Figure 1B). As a ‘positive’ control, exposure to EGTA (2 mM) resulted in a rapid FITC-dextran-4 flux from the basolateral side to the lumen, and the L/BL ratio was set to 1 (Figure 1B). Exposure of Caco-2 spheroids to both EO and EP (20 and 40 mM) increased the intraluminal FITC-dextran-4 fluorescence signals (Figure 1A and 1C) and significantly increased the L/BL fluorescence ratio versus the medium only control, in a dose dependent manner (*p*<0.0001; Figure 1B and 1D). Interestingly, the magnitude of the effects differed between EO- and EP-treated spheroids. The mean FITC-dextran-4 fluorescence ratio was significantly higher in response to 20 µM EO compared to 20 µM EP (0.34±0.02 vs. 0.21±0.03, respectively; *p*<0.0001) as well as in response to 40 µM EO compared to 40 µM EP (0.45±0.06 vs. 0.38±0.10, respectively; *p*<0.001) (Figure 1B, D).

### Effects of FAEEs on cell membrane integrity (LDH leakage)

**Figure 2:**
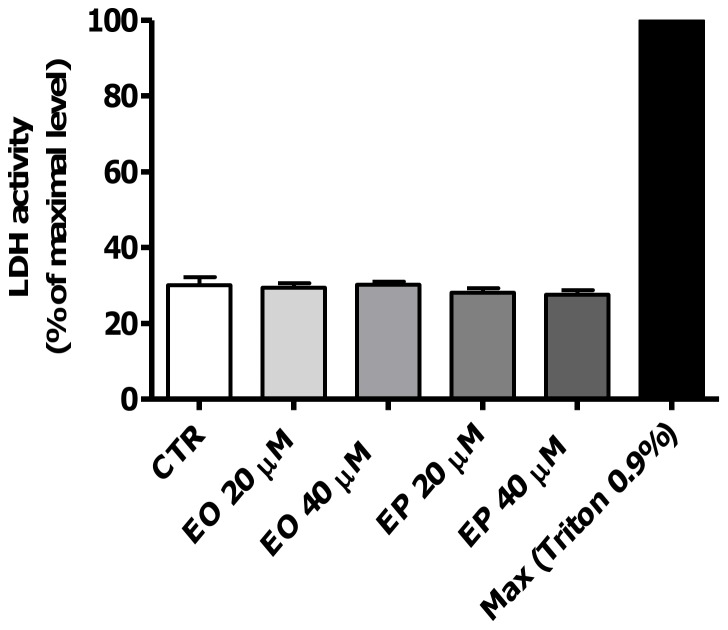
Effect of ethyl oleate (EO) and ethyl palmitate (EP) on LDH release in Caco-2 spheroids. Caco-2 spheroids were treated with a cell lysis buffer to induce maximum LDH leakage (Max), medium only as control(CTR), EO 20 and 40 µM or EP 20 and 40 µM at 37 °C for 24 h. LDH activity was determined by a fluorescent assay (n = 3). Data are reported as percentage of maximum LDH release and values are presented as means±SD, *p*>0.05 vs. medium only-treated control.

To investigate whether exposure to EO or EP can reduce cell viability of Caco-2 cells, cell membrane integrity was examined by measuring LDH release. No significant increase in LDH activity was detected after incubation with neither 20 µM nor 40 µM of EO or EP compared to medium only control (*p*>0.05, Figure 2).

### Effects of FAEEs on localization and protein levels of ZO-1 and occludin

**Figure 3:**
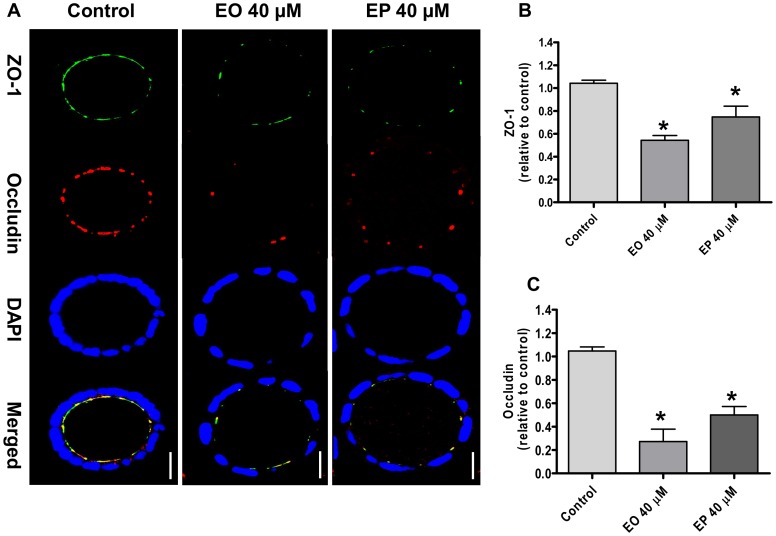
Effect of ethyl oleate (EO) or ethyl palmitate (EP) on ZO-1 and occludin localization in 3D Caco-2 spheroids. [**A**] Spheroids were exposed to medium only (control), EO 40 µM or 40 µM EP for 24 h, and immunostained for ZO-1 (green), occludin (red) and nuclei (blue) by confocal immunofluorescence staining, and representative images captured from the middle of spheroids are shown. The bar indicates 10 µm. **Exposure to ethyl oleate (EO) and ethyl palmitate (EP) decreases ZO-1 and occludin protein levels.** ZO-1 [**B**] and occludin [**C**] protein levels relative to control after exposure to medium only (control), EO 40 µM, or 40 µM EP for 24 h. *p<0.0001. ZO-1 and occludin protein levels were lower after EO 40 µM vs. EP 40 µM; (*p*<0.01 and *p*<0.05, respectively).

Since TJs are dynamic structures and respond quickly to pathophysiological stimuli, modulation of barrier function is often associated with changes in TJ integrity. Therefore, we used confocal immunofluorescence microscopy imaging to determine the effect of EO and EP on the intercellular protein localization of ZO-1 and occludin in Caco-2 spheroids. No changes occurred in localization of either ZO-1 or occludin in medium-only treated control spheroids (Figure 3A). By 24 hours, exposure to EO or EP resulted in loss of ZO-1 and occludin at the intercellular junctions and caused mislocalization of both proteins at the plasma membrane (Figure 3A). To evaluate whether the FAEEs-mediated changes in ZO-1 and occludin localization are accompanied by reduced levels of their proteins, a cell-based ELISA was used. In this assay, proteins levels are measured in fixed cells, eliminating the need for lysate preparation. Compared with medium only-treated control spheroids, significant reduction of ZO-1 and occludin levels were observed in EO-treated spheroids (*p*<0.0001; Figure 3B and 3C, respectively). Similarly, treatment with EP significantly reduce ZO-1 and occludin protein levels compared to that observed in medium only-treated controls (*p*<0.0001; Figure 3B and 3C, respectively). In line with the higher fluorescence ratio, EO 40 µM was also more potent in decreasing ZO-1 and occludin protein levels than EP 40 µM (*p*<0.01 and *p*<0.05, respectively; Figure 3B and 3C).

### Effects of FAEEs on cellular oxidative stress

**Figure 4:**
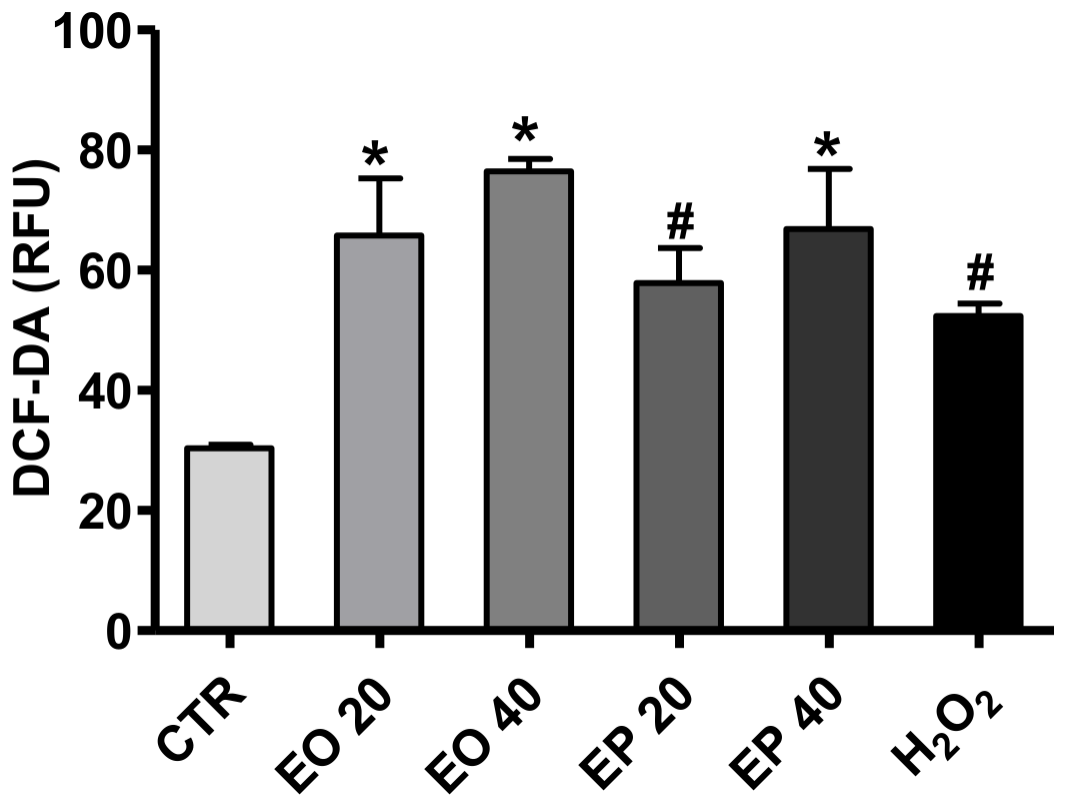
Effects of ethyl oleate (EO) or ethyl palmitate (EP) on ROS generation in the 3D Caco-2 spheroids. The spheroids were pretreated with 100 µM DCF-DA for 1 h at 37°C and subsequently with either medium only as negative control (CTR), EO 20 or 40 µM), EP 20 or 40 µM or H_2_O_2_ (30 µM) as positive control for 24 h. Cellular ROS production was measured by DCF fluorescence, and reported as relative fluorescent units (RFU). Data expressed as means±SD, *p<0.0001 and **^#^** *p*<0.001 vs. CTR.

DCF-DA was used to test whether FAEEs can induce ROS generation in Caco-2 spheroids. Exposure to 20 µM or 40 µM EO or EP for 24 h, dose-dependently increased the intracellular ROS contents of 3D spheroids compared to medium-only treated controls (*p<0.0001; Figure 4). ROS generation was higher in response to EO 20 µM and 40 µM compared to EP 20 µM and 40 µM, respectively. However, these differences did not reach statistical significance (both *p*>0.05; Figure 4).

### Effects of resveratrol on FAEEs-induced oxidative stress

**Figure 5:**
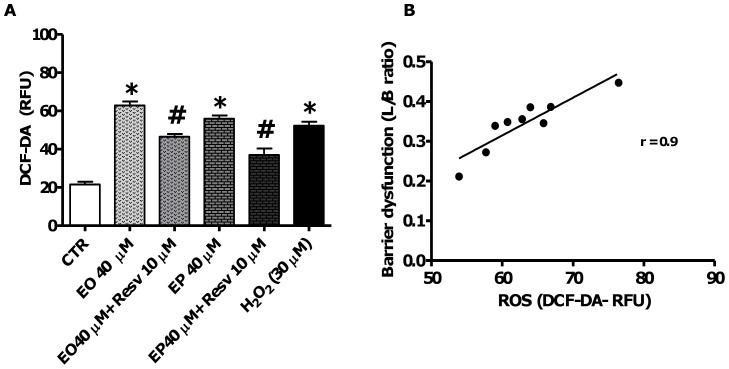
Effects of Resveratrol (Resv) on ethyl oleate (EO)- and ethyl palmitate (EP)-induced oxidative stress. [**A**] Spheroids were exposed to medium only as control (CTR) or 40 µM of either EO or EP for 24 h, or first treated with 10 µM resveratrol for 1 h prior to treatment with either EO or EP for 24 h. Values are means±SD, *p<0.0001 vs. CTR, ^#^ *p*<0.0001 vs. treated only with EO or EP. [**B**] The association between the increase in ROS generation and paracellular permeability was analyzed by Spearman test (*r* = 0.9 and *p*<0.01).

Resveratrol (3, 4′, 5 tri-hydroxystilbene), a polyphenol found in grape skin and red wine, has been shown to possess a wide range of biological and pharmacological properties including potent antioxidant activity by scavenging free radicals and inhibiting lipid peroxidation [29], [30], [31], [32]. Pretreatment of spheroids with 10 µM resveratrol prior to challenge with either 40 mM EO or EP, significantly attenuated the EO- and EP-induced increase in intracellular ROS levels (*p*<0.05; Figure 5A). This increase in ROS was higher in response to EO 40 µM compared to EP 40 µM, respectively (*p*<0.05; Figure 5A). Furthermore, a linear correlation was found between the increase in ROS generation and the decrease in paracellular barrier function after FAEEs exposure (*r* = 0.9 and *p*<0.01; Figure 5B).

### Involvement of ROS in FAEEs-induced changes in paracellular permeability

**Figure 6:**
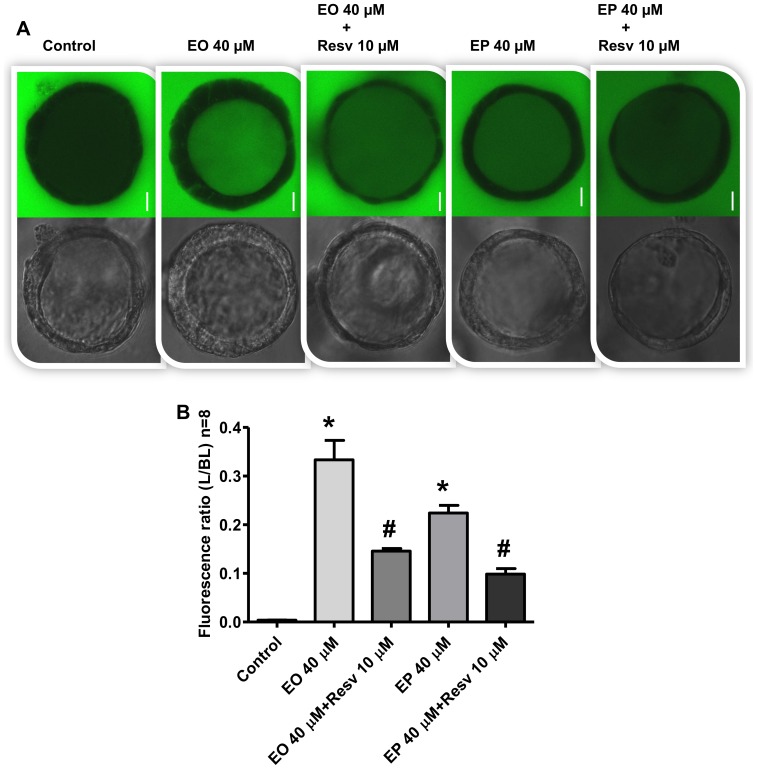
Effects of Resveratrol (Resv) on the ethyl oleate (EO)- and ethyl palmitate (EP)-induced barrier dysfunction in Caco-2 spheroids. Spheroids were exposed to medium (control) or treated with EO or EP for 24 h, or first treated with 10 µM resveratrol for 1 h prior to treatment with 40 µM of either EO or EP for 24 h. [A] Intraluminal accumulation of FITC-dextran-4 (green) was observed using confocal microscopy and representative images captured from the middle of spheroids are shown. The bar indicates 10 µm. [B] The mean fluorescence intensity of FITC-dextran-4 after exposure was measured and expressed as the ratio of the luminal (L) over the basal (BL) compartment. Values are Means±SD, *p<0.0001 vs. medium only-treated control, ^#^ *p*<0.0001 vs. treated only with EO or EP. FITC-dextran-4 fluorescence ratio, EO 40 vs. EP 40 µM; (*p*<0.0001, Figure 6A and 6B).

To investigate the role of oxidative stress and increased H_2_O_2_ as mediator of the FAEEs-induced increase in epithelial permeability, Caco-2 spheroids were pretreated with 10 µM resveratrol at 37°C for 1 h, followed by incubation with 40 µM EO or EP for 24 hrs. Exposure of Caco-2 spheroids to EO increased the intraluminal FITC-dextran-4 flux (Figure 6A), and significantly increased the L/BL fluorescence ratio compared with the medium-only treated controls (*p*<0.0001; Figure 6B). Resveratrol attenuated EO-induced FITC-dextran-4 flux (Figures 6A), and significantly decreased L/BL fluorescence ratio compared to EO alone (*p*<0.0001; Figure 6B). A similar attenuating effect of resveratrol was observed for EP-induced barrier dysfunction (*p*<0.0001; Figures 6A and 6B).

### Role of ROS in FAEEs-induced changes in localization and protein levels of ZO-1 and occludin

**Figure 7:**
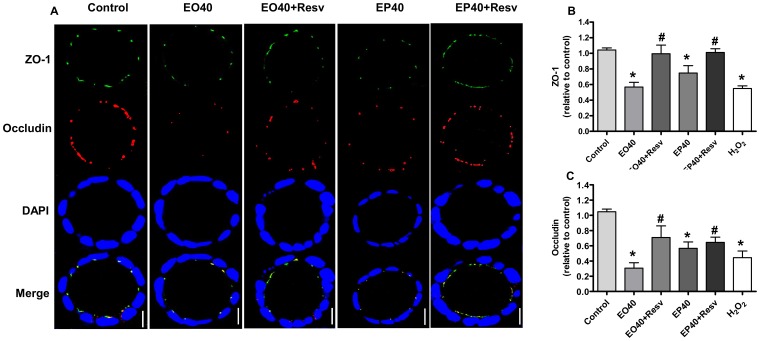
Effects of Resveratrol (Resv) on the ethyl oleate (EO)- and ethyl palmitate (EP)-induced-induced changes in ZO-1 and occludin localization, and protein levels. [A] Spheroids were exposed to medium (control) or treated with 40 µM of either EO or EP for 24 h, or first treated with 10 µM resveratrol for 1 h prior to treatment with either EO or EP for 24 h, and immunostained for ZO-1 (green), occludin (red) and nuclei (blue) by confocal immunofluorescence staining, and representative images captured from the middle of spheroids are shown. The bar indicates 10 µm. [B] ZO-1 and [C] occludin protein levels relative to control after exposure to the indicated concentrations of EO, EP and H_2_O_2_. Values are Means±SD, *p<0.0001 vs. medium only-treated control, ^#^ *p*<0.0001 vs. treated only with EO or EP. ZO-1 and occludin protein levels, EO 40 µM vs. EP 40 µM; both (*p*<0.05).

The effects of EO and EP on ZO-1 and occludin localization in Caco-2 cell spheroids were analyzed in either presence or absence of resveratrol pretreatment. Control spheroids showed apical intercellular localization of ZO-1 and occludin with strong co-localization of both proteins in the tight junctions (Figure 7A). EO and EP treatment resulted in reduced staining for both ZO-1 and occludin with miscolocalization of both proteins. The disruption of ZO-1 and occludin induced by FAEEs was partially prevented by resveratrol (Figure 7A). Pretreatment of the spheroids with 10 µM resveratrol for 1 h was able to significantly preserve ZO-1 and occludin proteins levels, compared to EO alone (*p*<0.0001; Figure 7B and 7C, respectively). Similarly, pretreatment of the spheroids with resveratrol significantly preserved the ZO-1 and occludin protein levels compared to EP (*p*<0.0001; Figure 7B and 7C, respectively). No differences could be observed in ZO-1 and occludin protein levels between EO and EP after pretreatment with resveratrol (*p*>0.05; Figure 7B and 7C, respectively).

### Effects of FAEEs on intracellular ATP levels

**Figure 8:**
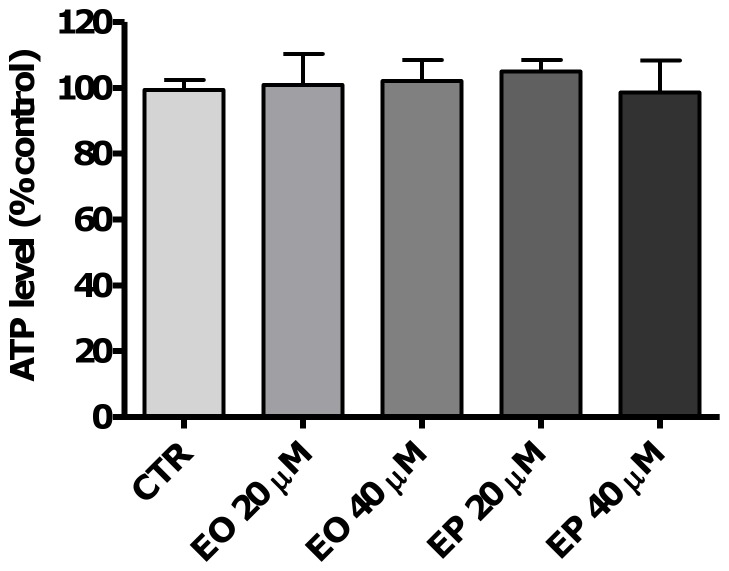
Effects of ethyl oleate (EO) and ethyl palmitate (EP) on intracellular ATP levels. Caco-2 spheroids were treated with medium only as negative control (CTR), EO 20 or 40 µM), EP 20 or 40 µM or H_2_O_2_ (30 µM) as positive control for 24 h. Intracellular ATP levels were determined by a bioluminescence assay (n = 3). Data are reported as percentage of untreated controls and values are presented as means±SD, *P*>0.05 vs. CTR.

As TJs stability is influenced by a number of intracellular events including energy depletion [33], we assessed whether FAEEs can reduce intracellular ATP levels in Caco-2 spheroids. As shown in Figure 8, exposure to 20 µM or 40 µM of either EO or EP for 24 h did not decrease the intracellular ATP levels compared to control (100.9±9.4% and 102.1±6.4%, 104.9±3.6 and 98.5±9.8, respectively vs. 102.2±4.0%, *p*>0.05). There were also no differences in the intracellular ATP levels between EO and EP treatments.

## Discussion

This study is the first to show that the non-oxidative ethanol metabolites ethyl oleate (EO) and ethyl palmitate (EP) cause a dose-dependent disruption of the intestinal epithelial TJs, resulting in increased paracellular permeability without compromising cell viability. Furthermore, EO and EP increased cellular oxidative stress, which was found to correlate with barrier dysfunction. Preincubation with the antioxidant resveratrol ameliorated the FAEEs-induced TJ disruption and barrier dysfunction.

The interaction between FAEEs and intestinal epithelial cells has been examined using a Caco-2 cell 3D culture model, which presents a physiological *in vitro* model to study (patho) physiological functions of intestinal epithelial cells, including morphogenesis [23] and TJs integrity [24]. Our data showed that treatment of epithelial cells with 20 or 40 µM of either EO or EP results in enhanced paracellular permeability in a dose-dependent manner. The concentrations we used are within the biologically relevant, physiological range attained in serum (20–40 µM) after ingestion of a drink equivalent to 10 g ethanol [34].

An increase in paracellular permeability can be caused by different events including epithelial cell death [35]. However, cellular viability was not compromised in the present study, indicating that the FAEEs-induced barrier dysfunction was independent of loss of cell viability. Cytotoxic effects of FAEEs have been reported on pancreatic [36] and liver cells [37], but in those experiments much higher concentrations of FAEEs (100–800 µM) have been used.

As disruption of the TJs will contribute to changes in permeability, we examined whether the FAEEs-induced barrier dysfunction could be due to changes in localization an expression of TJ proteins. Exposure to FAEEs did alter ZO-1 and occludin localization and decrease their protein levels, indicating that changes in paracellular permeability observed by FAEEs treatment were due to disruption of TJs integrity. Our data confirm previous findings by our group [24], and by others on ethanol and the oxidative metabolite, acetaldehyde [4], [7], [8], [9], [11], [38], [39]. However, we expand on these observations by showing for the first time that these changes can also be induced by the non-oxidative metabolites FAEEs, and thus may contribute to barrier dysfunction after ethanol ingestion.

Mislocalization and/or internalization of the TJ proteins can result in rapid changes in the composition and structure of the TJ proteins [40], [41]. Intracellular interaction between occludin and actin filaments through ZO-1 plays an important role in organization of the TJs [42]. In this study, no intracellular staining of ZO-1 or occludin was observed, suggesting involvement of a process of disassembly rather than active remodeling through internalization. Moreover, our cell-based ELISA revealed reduced ZO-1 and occludin protein levels, most likely due to inhibition of protein synthesis. In line with these data, FAEEs have also been shown to inhibit protein synthesis in acinar cells [43] and hepatocytes [21].

Although FAEEs have been shown to induce ROS generation in hepatocytes [44] and blood cells [45], no such data were available with respect to intestinal cells. In this study, exposure to EO and EP dose-dependently increased ROS generation in Caco-2 spheroids. Furthermore, ROS production was found to correlate with the FAEEs-induced barrier dysfunction. Previously, an increase in oxidative stress has been shown to disrupt mucosal barrier function of intestinal epithelial cells through mechanisms involving oxidation, nitration, disassembly, and instability of the actin cytoskeleton [46], [47], and cellular microtubules [24]. In addition, increased ROS production has been implicated in membrane damage and the subsequent amino acid transport reduction that is necessary for protein core synthesis [48], which may also contribute to the observed decrease in ZO-1 and occludin protein levels.

Oxidative stress-mediated intestinal barrier disruption may offer opportunities for intervention strategies with antioxidants. Therefore, we tested whether resveratrol at nutritionally relevant concentrations can attenuate FAEEs-induced barrier function. The main reason for reduction of oxidative stress by resveratrol is its ability to scavenge intracellular ROS [49]. Since resveratrol is effectively metabolized by Caco-2 cells, enterocytes are considered a major target site for this dietary antioxidant [50]. Pretreatment with resveratrol attenuated the FAEEs-induced ROS generation, barrier dysfunction and changes in ZO-1 and occludin. Taken together, these findings suggest that the FAEEs-induced barrier dysfunction is oxidative stress-dependent. These observations are in line with previous data demonstrating that antioxidants can inhibit ROS (H_2_O_2_)-induced paracellular hyperpermeability [51]. In the present study, FAEEs increased H_2_O_2_ production, which has previously been found to increase paracellular permeability in Caco-2 monolayers via protein kinase C- [52], mitogen activated protein kinase- [52] and protein tyrosine-dependent mechanisms [53]. Involvement of these signaling pathways in the FAEEs-induced ROS and modulation of barrier dysfunction cannot be ruled out, and merits further investigation.

Oxidants have been shown to deplete ATP in different cells and such changes in intra-cellular ATP levels are known to affect epithelial permeability [54], [55]. However, in this study EO and EP failed to influence the intracellular ATP levels. In contrast, Criddle *et al*. have demonstrated that FAEEs, namely palmitoleic acid ethyl ester (100 µM), can inhibit ATP synthesis in pancreatic acinar cells [22]. The discrepancy between these results and our observations may in part be explained by the difference in the type of the FAEEs and the concentrations employed.

In this study, EO showed a stronger effect than EP in increasing the FITC-dextran-4 flux in Caco-2 spheroids. EO was also found to induce a more pronounced decrease in ZO-1 and occludin protein levels and a higher increase of ROS compared to EP. Given the higher concentrations of serum EO in long-term alcoholics and binge drinkers (9865 and 557 pmol/ml, respectively) [56], these observations indicate that EO generated after ethanol consumption is the main contributor of FAEEs induced barrier dysfunction *in vivo*. Since EP can also be detected in plasma and lead to barrier disruption, the presence of both metabolites may have additive or even synergetic injurious effects on intestinal epithelial integrity.

It is noteworthy that Caco-2 cells are transformed cells, raising the possibility that the effect of ROS in a nontransformed cell may be different. However, the transformed cells are known to be resistant to ROS-induced injury [53], and therefore, ROS may be more injurious to the intestinal epithelial than the observations in this study. *Ex vivo*, inhibition of the oxidative ethanol metabolism has been shown to result in shifting ethanol metabolism towards the non-oxidative pathways resulting in FAEEs generation [57]. Therefore the damaging effects of FAEEs are expected to be particularly important in chronic alcohol users with low ADH activity. In heavy drinkers, FAEEs have been found to remain elevated for up to 99 hours [14]. Based on the present data, it can be speculated that accummulation of FAEEs in alcoholics may contribute to the pathogenesis of alcoholic liver disease by inducing intestinal barrier disruption and consequently liver injury. Therefore, our study provides a scientific rationale to further investigate the role of FAEEs in ethanol-induced gut ad liver diseases in humans.

In summary, our study shows for the first time that FAEEs are able to induce intestinal barrier dysfunction partly by ROS-induced TJs modulation and thereby may contribute to the pathogenesis of ALD. Furthermore, our results demonstrate that resveratrol in intestinal epithelial cells can combat the damaging effects of FAEEs-induced oxidative stress on mucosal barrier function. Our findings a) provide new insights into understanding the role of the non-oxidative metabolism in ethanol-induced intestinal injury, which may be especially relevant in chronic alcoholics and b) indicate the involvement of oxidative stress which might have the potential to test antioxidants as therapeutic target to reduce the noxious effects of ethanol on the small and large intestine.
